# The great nursing brain drain and its effects on patient safety

**DOI:** 10.1186/s13756-020-00719-4

**Published:** 2020-04-30

**Authors:** Alexandra Peters, Rafael Palomo, Didier Pittet

**Affiliations:** 1 Infection Control Programme, University of Geneva Hospitals and Faculty of Medicine, Geneva, Switzerland; 2 University of Geneva, Geneva, Switzerland

**Keywords:** Infection prevention and control, Infection control, Hand hygiene, Nurses, Midwives, World Health Organization

## Abstract

The World Health Organization (WHO) has declared 2020 the “Year of the Nurse and Midwife”. On May 5th of this year, for the annual celebration of the SAVE LIVES: Clean Your Hands campaign, the WHO will focus on the critical role of nurses and midwives in promoting public health. The brain drain of healthcare workers (HCWs) leads to unequal distribution of nurses and midwives around the world, which affects the quality of care provided to patients. This phenomenon should be addressed as a global problem as it highlights staff shortages in the health system.

## Introduction

The 5th of May is the international day of hand hygiene and the annual World Health Organization (WHO) “SAVE LIVES: Clean Your Hands” campaign. As 2020 is the WHO year of the Nurse and Midwife, the hand hygiene campaign will also center around the importance of nurses and midwives to public health.

Nurses and midwives are a major group of healthcare workers (HCWs) who are essential to health systems considering their frequent contact that with patients [1]. This vital role involves great responsibility: they are performing the most interventions on patients in order to save their life, that puts them at higher risk of making a mistake which might have serious consequences. The issues around the global migration of nurses and midwives, generally from poorer or less stable to richer or more stable countries, has a profound effect on the way health systems can be organized and ultimately on the quality of patient care.

The so-called “brain drain” of HCWs is a phenomenon that should be taken seriously. The flow is global and constant, and partially explains why countries like Switzerland have 17.3 nurses and midwives per 1000 inhabitants (2016) compared to 0.3 in Senegal the same year and 0.4 in Mozambique (2017) [1]. Every year, thousands of workers leave their country because of a lack of professional opportunities, low income or the presence of an armed conflict. These reasons are considered as “push factors”. Added to this, countries attracting these workers have “pull factors” such as a large offer of jobs with favorable economic conditions [2]. The countries of departure are mainly poor states but not only. Sub-Saharan Africa suffers in particular. In 2013, 70% of students in the health field who graduated in Guyana migrated to work in another country [2]. In Tanzania, Mozambique and Liberia, the states recorded the departure of more than half of the newly trained staff in the same year [2]. This phenomenon also exists between rich countries and within a country. For example, the rather urban cantons of Zurich and Basel have a higher concentration of nurses than rural cantons such as Uri in Switzerland [2].

The WHO estimates that a density of less than 23 HCWs per 1000 habitants is not compatible with a viable health system. Since nurses and midwives represent half of the staff, it can be assumed that a density of less than 11.5 nurses and midwives per 1000 population is not compatible with the standard of quality sought by WHO [2]. The brain drain results in an unequal distribution of nurses and midwives around the world, which weakens neglected health systems and, ultimately, affects the quality of care provided to the patient [2, 3].

### The brain drain is associated with a decrease in the quality of care

Many studies have analyzed the relationship between the quality of care provided and the patient load of caregivers. A study conducted in the United States statistically demonstrates that an increase of nurses’ workload was associated with a decrease of patient satisfaction, a worsening of outcomes and an increase of nosocomial infections. In other words, the more a nurse must deal with a large number of patients, the more the quality of care is affected [4]. The brain drain of nurses also has indirect consequences on the quality of care in multiple areas. The study describes the variables concerned, the sum of which, cause the deterioration of care. If nurses have more patients, they can logically spend less time on each one. The consequence of this is that certain important but time-consuming interventions cannot be carried out. In the context of a work overload, the study observed that 8 to 10% of nurses committed “violations” during routine situations and between 32 and 53% during emergency situations. These “violations” are defined when the caregiver deviates from mandatory procedures. When overworked, nurses and midwives have less time to communicate with the patient and with colleagues. Such communication is however crucial for the overall management of a patient. Secondly, such a stressful work environment promotes the loss of motivation which most often results in reduced efficiency and absenteeism. Overall, these issues aggravate the situation by further reducing the number of nurses who can work [4].

Another systematic study looking at the nurse-to-patient ratio found that the larger the healthcare teams were, the fewer medical errors were made and also resulted in a lower mortality among patients [3].

### The brain drain in a context of shortage

In 2016, the WHO published a study that showed a shortage of HCWs worldwide. Indeed, there are a shortage of 9 million nurses and midwives worldwide. When we look at the distribution of the shortage of caregivers, we notice with regard to nurses and midwives that about 70% of the 9-million-person shortage concerns lower middle and low income countries [5]. Thus, the countries suffering the most from the brain drain are paradoxically also those which lack the most caregivers, and have more fragile health systems. To make matters worse, regions suffering from brain drain often have a higher burden of disease than the others benefiting from it [2].

The WHO has defined projects to encourage an increase in the training of health professionals. This intervention if implemented effectively, should gradually decrease the number of missing nurses and midwives. But the situation becomes even more alarming if one observes the projections of the WHO for 2030. Although projections indicate an improvement in most of rich countries, low-income countries and in particular Africa will see their shortage worsen. The projections show that the number of nurses and midwives missing on the African continent will increase from 1.8 million to 2.8 million [5].

This migration is of course not the primary cause of the shortage itself in developing countries. However, it contributes to worsening the already dire situation of certain states which already have a fragile health system. This precariousness increases the desire or the need of HCWs to leave and results in the brain drain. We are therefore facing a vicious circle [5].

### Retain and not attract

In its 2016 report on the workforce required in the health sector, the WHO concludes with a recommendation to apply economic and social measures that would prevent the depletion of HCWs in the low-income countries. These measures concern both the countries of departure and the countries of arrival, and it is well-recognized that progress can only be made if comprehensive and united measures are put in place [5].

The Organization for Economic Cooperation and Development (OECD) published an article about this specific topic in 2010 [6]. It highlights the importance of pursuing the ideal of a balanced of training and retention to address the need for HCWs on a national level. Potential measures would mainly concern the countries of arrival, which would thus decrease the quantity of places available for immigrants, and thus these countries would become less attractive destinations. These same countries should also commit financially to the countries of departure to support the development of the health system, including improvement of working conditions and personnel management, which in turn would help to reduce the “push factors” [2, 6].

Another article published in 2013 in a Swiss journal also proposed the implementation of methods making it more difficult for nurses and midwives to leave their home country permanently [2]. Training abroad in a country where the education system is internationally recognized only benefits the country of departure if the trained personnel return. It was therefore proposed to put a strict limit on the length of stay (work permit / study) in the countries that were training HCWs. This is of course not always in the interest of the countries that train the workers, as they have a social and economic interest to keep educated immigrants that they have trained [2, 6].

It is important to situate the brain drain of nurses and midwives in the context of migration affecting all professional sectors. The unequal living conditions between countries push workers from all fields to migrate. This implies that any comprehensive approach must be aimed at an overall improvement in the standard of living for people in their country of origin. In other words, addressing the underlying causes of social and economic inequality is probably the central challenge to be tackled.

## Conclusion

The migration of nurses and midwives belongs to both the causes and the consequences of the deterioration of health systems, and low-income countries suffer the most. These mechanisms and the motivations that feed them must be carefully studied if we do not want to contribute to the marked polarization and increased inequality between the various health systems in the world. This migratory phenomenon is symptomatic of a global context of staff shortages in the health system, and if we don’t address this as a global problem, we risk worsening its consequences.

## Data Availability

Data sharing not applicable to this article as no datasets were generated or analyzed during the current study.
